# Emotion regulation strategies modulate the effect of adverse childhood experiences on perceived chronic stress with implications for cognitive flexibility

**DOI:** 10.1371/journal.pone.0235412

**Published:** 2020-06-26

**Authors:** Vrinda Kalia, Katherine Knauft

**Affiliations:** Department of Psychology, Miami University, Oxford, Ohio, United States of America; University of Jyvaskyla, FINLAND

## Abstract

Exposure to early life adversity is associated with chronic stress and a range of stress-related health problems in adulthood. Since chronic stress debilitates activity in the prefrontal cortex (pFC), maladaptive regulatory strategies in response to stress have been proposed as one explanation for the impact of early life adversity on health outcomes in adulthood. We conducted a study to examine the impact of adverse childhood experiences (ACEs) on cognitive flexibility, a key executive function implicated in activity in the pFC, in a sample of adults (N = 486). Additionally, we investigated whether perceptions of chronic stress in adulthood would mediate the influence of ACEs on cognitive flexibility. However, stress is a subjective experience, and emotion regulation strategies can attenuate the stress response. So, we also examined if individual differences in emotion regulation strategies would modulate the relationship between ACEs and chronic stress. Our results demonstrate that early life adversity, as characterized by ACEs, is associated with decreased cognitive flexibility in adulthood. Additionally, number of ACEs was positively correlated with perceived stress, which in turn was negatively correlated with cognitive flexibility. But, individual differences in the habitual use of emotion regulation strategies moderated the influence of ACEs on chronic stress. Specifically, habitual use of cognitive reappraisal attenuated the stress levels whereas habitual use of expressive suppression exacerbated stress levels. Overall, our study highlights the importance of examining emotion regulation in individuals who have experienced early life adversity.

## Introduction

Exposure to adverse experiences in early development is associated with increased risk of mental illness and negative health outcomes in adulthood [1]. For instance, exposure to adversity in childhood is correlated with increased risk for depression in adulthood [2]. Early life adversity (ELA; i.e., exposure to environmental experiences that deviate from normative stimulation that fosters brain development) has also been implicated in increased risk for heart disease and coronary distress [3, 4]. Considering that approximately two-thirds of Americans report exposure to some form of ELA [5], this places a major burden on the public health system. Thus, it is relevant to examine factors and variables that enhance or attenuate the impact of ELA on health outcomes in adulthood.

Both positive and negative experiences in early development have a profound impact on the brain’s development [6]. ELA, such as abuse, parental neglect, or deprivation, can alter the normative development of neural circuitry [7]. Neurobiological evidence has shown that ELA can have a debilitating impact on prefrontal cortex (pFC) structure and function [8, 9] in particular. Since cognitive control and strategic thinking are implicated in the pFC [10], this can negatively influence goal-directed behavior in adulthood. Yet, the putative implications of ELA on cognitive and emotional regulatory capacities, such as cognitive flexibility, that are implicated in activity in the pFC are understudied [1].

In addition to having a negative impact on pFC development, ELA is associated with enduring changes in the central nervous system [3]. ELA occurs at a time when the hypothalamic-pituitary-axis (HPA axis) is still immature, and exposure to increased levels of stress hormones can disrupt normative development of the structure (e.g., reduced volume of the hippocampus) and function (e.g., persistent overactivation) of the HPA axis [11]. As a result, ELA can unbalance the normally adaptive physiological stress response [12], which disrupts the body’s ability to maintain stability in the face of changing environmental demands (or allostasis) and results in structural and functional abnormalities in brain development [13]. Consequently, the short-term stress response initiated adaptively to environmental cues may become an enduring activation of the stress systems with detrimental consequences [13]. One proposed explanation for the association between ELA and physical morbidity in adulthood is the habitual deployment of maladaptive coping strategies (e.g., drug use) in response to stressful events [5]. However, not all individuals exposed to ELA exhibit negative outcomes associated with early life stress. Individual differences in emotion regulation could account for some of the observed variance in outcomes [14, 15].

### Emotion regulation strategies

People attempt to regulate their emotions in myriad ways. Gross’s process model of emotion regulation suggests that emotion regulation begins after the identification of an emotion as being helpful or harmful to a goal [16]. Emotion regulation strategies help to maintain, increase, or decrease an individual’s response to emotion-evoking events [17]. Gross [18] has identified two key strategies–expressive suppression and cognitive reappraisal.

Expressive suppression is a strategy in which an individual attempts to conceal or inhibit emotional expressions [18]. It is important to note that this strategy fails to change the emotional experience. Instead, experimental evidence indicates that it may enhance physiological response associated with the emotion [18]. Longitudinal work has shown that habitual use of expressive suppression is associated with more intrapersonal costs, such as increased fatigue and lower self-esteem [19]. Additionally, a meta-analytic review of the literature on the relationship between emotion regulation strategies and psychopathology has demonstrated that suppression is associated with increased levels of psychopathology [20]. There are some studies that indicate that the cost of suppression is dependent on factors such as how burdensome [21] or inauthentic [22] it feels to suppress one’s feeling. Regardless, suppression is viewed overall as a costly emotion regulation strategy that is associated with negative health outcomes [23, 24].

Reappraisal, on the other hand, requires that the individual alter or update the way they think about or frame an event to enhance or reduce its emotional impact [25]. In order to engage in reappraisal as an emotion regulation strategy, an individual has to intentionally shift one’s appraisal of the situation or the goal that’s associated with the emotion. Gross and colleagues have shown that, in comparison to suppression, reappraisal is an adaptive method for regulating one’s emotions [18]

### Early life adversity, emotion regulation, and chronic stress

Reappraisal is particularly germane within the context of a stress response. Stress is a subjective experience that engages both the brain and the body [8]. The physiological stress response emerges from an interaction of individual characteristics and the environmental context [13]. The biopsychosocial model of challenge and threat [26] provides an account of individual differences in the experience of stress by focusing on the person’s appraisal of available resources. When demands exceed available resources, the situation is appraised as threatening. But, when one perceives they have sufficient resources to meet contextual demands, the stressor is evaluated as challenging, not threatening [26]. In effect, an individual’s stress response is dependent on the way they appraise a stressor.

Early life stressors, however, which could take the form of maltreatment (physical or sexual abuse) or neglect, often exceed the child’s ability to evaluate or cope [1]. These early stressors are more likely to be perceived as uncontrollable and hence more debilitating [27]. As a result, the individual might develop short-term or maladaptive coping strategies (e.g., drug use) that help them cope with their stress [5]. These maladaptive behaviors, in turn, account for the link between early life stress and negative health outcomes [3]. Indeed, converging evidence indicates that neurobiological development in response to ELA may be the mechanism that results in cognitive and emotional impairment thereby increasing risk of a variety of stress-related health problems [5].

However, there is also some evidence to suggest that individuals exposed to ELA exhibit enhanced cognitive skills in ecologically relevant tasks associated with threat and danger [28]. Additionally, individuals exposed to ELA appear to develop independent emotion regulation strategies faster than those who have not been exposed to ELA [7]. This raises the possibility that individual differences in emotion regulation could influence the association between ELA and chronic stress experienced in adulthood. Thus, it is possible to speculate that individuals with adaptive emotion regulation strategies might be able to attenuate the impact of ELA on pFC-regulated cognitive abilities.

### Current study

We conducted a study to investigate the effect of ELA on cognitive flexibility in adulthood. We conceptualize ELA as the enduring soil that houses the seeds for cognitive and emotional outcomes in adulthood. We assessed ELA using the Adverse Childhood Experiences Scale (ACEs). Cognitive flexibility is a key component of executive functions [29] that is often the last to emerge in development [30]. It is a multifaceted construct that exhibits both trait and state characteristics [31]. Researchers have approached and measured cognitive flexibility in several different ways [32]. Some researchers propose that set shifting and attention shifting are key characteristics of cognitive flexibility [30]; whereas other researchers have highlighted the flexible adaptation of behavior and cognitive processes to changing environmental demands [31]. One outcome associated with cognitive flexibility is that it allows the individual the ability to reframe and reconsider information in a challenging context to enable effective coping [33].

Depending on their approach, tasks measuring cognitive flexibility assess different aspects of cognitive flexibility [34]. For example, the Wisconsin Card Sort Test (WCST) [35], which is behavioral measure of cognitive flexibility, isolates an individual’s ability to shift between rules as a way to determine cognitive flexibility. Self-report measures of cognitive flexibility, which offer a practical alternative to behavioral measures, can be used to provide insight about state characteristics of cognitive flexibility [36]. In this study, we focused on the kind of cognitive flexibility that is highlighted in cognitive behavioral therapeutic settings [34]. Specifically, our goal was to assess the individual’s ability to reformulate and restructure maladaptive thoughts in response to challenging circumstances and affective states [34]. In order to do so, we assessed cognitive flexibility using the Cognitive Flexibility Inventory (CFI) [34].

We also sought to determine whether perceived chronic stress mediated the relationship between ELA and cognitive flexibility. Based on prior research showing that chronic psychosocial stress disrupts pFC functioning, which has a negative impact on attentional control in set-shifting tasks [37], we assumed that chronic stress levels would influence cognitive flexibility negatively. But, we also expected that chronic stress would mediate the relationship between ELA and cognitive flexibility because of the known impact of ELA on allostasis [13]. One of the physiological consequences of ELA is that it alters *allostasis*, which is the body’s ability to maintain stability through changes wrought by managing environmental demands [13]. This can result in an extreme or extended activation of the physiological stress response and is known as toxic chronic stress [13]. As a result, we expected that individuals with higher number of ACEs would also report increased stress levels [38]. This chronic stress has been proposed as the explanation for the association between ELA and negative health outcomes [39]. Since chronic stress reduces cognitive abilities implicated in the pFC [27], we speculated that ELA, partially through chronic stress, would have a negative impact on cognitive flexibility. Since stress is an individualized experience, we chose to focus on perceived chronic stress, which was measured using the Perceived Stress Scale (PSS) [40].

In contrast to its debilitating effect on cognitive abilities [41], some researchers have proposed that exposure to ELA might hasten emotion learning in development in some individuals [7]. Empirical evidence in support of this notion indicates that physically abused children are more likely to over-recognize angry or negative facial expressions [28, 42]. Hence, abused children may be more prone to interpreting neutral faces as angry or threatening. In effect, ELA might reprioritize development in the emotional systems in ways that offer advantages for survival [7].

Appraisal theories of emotion regulation raise the possibility that individual differences in emotion regulation processes could emerge as a key moderator of the impact of ELA on adult outcomes [43]. Specifically, appraisal theories highlight that it is the way an individual appraises an event that causes a stressful emotional reaction, not the event itself [43]. Thus, it is possible that in individuals with enhanced emotion regulation strategies we would observe an attenuated effect of ELA on perceived stress, and through perceived stress, on cognitive flexibility. Consequently, we also explored whether emotion regulation strategies would moderate the observed associations between ELA and perceived stress. We measured habitual use of emotion regulation strategies using the Emotion Regulation Questionnaire (ERQ) [17].

Based on prior research showing that exposure to ELA is associated with diminished cognitive flexibility [41, 44], our primary prediction was adverse childhood experiences (ACEs) would predict reduced cognitive flexibility (i.e., lower scores on the CFI). Because ELA disrupts the body’s ability to maintain stability in the face of changing environmental demands (or allostasis) [13], our second hypothesis was that individuals with higher number of ACEs would also report higher levels of stress. In other words, number of ACEs would be positively correlated with perceived stress levels. As chronic stress is associated with reduced cognitive flexibility [37, 45], our third hypothesis was that individuals with high levels of stress would also have lower scores on cognitive flexibility. Since previous research evidence indicates stress reactivity mediates the relationship between ELA and goal-directed behavior [46], our fourth hypothesis was that the relationship between ACEs and decreased cognitive flexibility would be mediated by the perceived stress. Considering that greater perceived stress has been shown to be associated with reduced habitual use of cognitive reappraisal [47], our fifth hypothesis was that reappraisal would attenuate the effect of ELA on perceived chronic stress, with implications for cognitive flexibility. Past research has shown that habitual use of suppression enhances the experience of perceived stress [48], so our final hypothesis was suppression would enhance the effect of ELA on perceived chronic stress and therefore exacerbate the impact of perceived chronic stress on cognitive flexibility.

## Methods

All study procedures were approved by the Institutional Review Board at Miami University; protocol # 01620. Written consent was obtained from all participants in the study. Following informed consent, participants completed measures assessing their exposure to adverse childhood experiences, perceived stress, and cognitive flexibility, in addition to demographics.

### Participants and procedure

Individuals living with the United States were recruited via Amazon’s Mechanical Turk (MTurk; N = 486; participants who failed more than half of the attention checks were removed; Male = 335; Female = 150; Missing = 1). Amazon’s Mechanical Turk (MTurk) is a web interface that allows researchers to recruit participants and conduct studies online. To initiate a study using MTurk, researchers must create an account with adequate funds to cover participant costs and any additional fees charged by Amazon. Participants responded to a brief description of the study and were recruited until the maximum number approved by the institutional review board was reached. They were compensated one dollar for their participation. The target sample size was determined using Monte Carlo simulations anticipating small to medium effect sizes. Participant age ranged from 19 years to 86 years (*M*_*Age*_ = 33.10, *SD* = 9.66; n = 77 participants did not provide age information). Majority of the participants self-identified as White (57.2%), and the remaining participants categorized themselves as Asian (22%), Hispanic (10.1%), African American (9.5%), and Native American (3.3%), Indian American (2.5%), race or ethnicity not listed (0.60%), or preferred not to disclose (0.40%).

### Measures

#### Adverse Childhood Experiences scale (ACEs) [49]

Consists of 10 questions assessing an individual’s exposure ELA including trauma and abuse. To capture the experience of early adversity, all questions regarding the ACEs pertain to the first 18 years of an individual’s life [50]. Five items in the scale ask about exposure to different types of maltreatment (e.g., *Did a parent or other adult in the household often push*, *grab*, *slap or throw something at you*?). The other five items request information about parental or family incapacities (e.g., *Were your parents ever separated or divorced*?). Every response in the affirmative (‘yes’) to a question was given 1 point. Although the exact definition of ELA continues to be debated [51], and different types of ELA (e.g., physical abuse, neglect) have traditionally been studied separately, empirical evidence indicates that they are interrelated and frequently co-occur [52, 53]. Additionally, prior research with ACEs has demonstrated a graded effect such that exposure to different types of ELA increases the risk for negative health outcomes [3, 54]. To account for the cumulative effect of ELA, we summed each individual’s yes responses to calculate their ACE score. Consequently, higher ACE scores indicate greater exposure to early adversity (α = 0.88).

#### Emotion Regulation Questionnaire (ERQ) [17]

Consists of 10 items, six that assess habitual use of cognitive reappraisal (e.g., *When I want to feel more positive emotion such as joy or amusement I change what I’m thinking about*; α = .87*)*, and four items assessing habitual use of expressive suppression (e.g., *When I am feeling positive emotions*, *I am careful not to express them*; α = .81). Each response is made on a seven-point Likert scale (1 = *strongly disagree* to 7 = *strongly agree)* and items for the two subscales are summed separately.

#### Perceived Stress Scale (PSS) [40]

Consists of 10 items that measure an individual’s perceived stress over the past month. Questions assess how overloaded individuals have generally felt (*e*.*g*., *In the last month*, *how often have you felt nervous and “stressed”*?*; In the last month*, *how often have you found that you could not cope with all the things you had to do*?). Responses are measured on a five-point Likert scale (*never* to *very often*). Items were reverse scored when necessary and summed such that higher scores indicated relatively more perceived stress (α = 0.86).

#### Cognitive Flexibility Inventory (CFI) [34]

Consists of 22 items that provide information about two types of cognitive flexibility: 1) Alternatives (the ability to perceive multiple alternate explanations and solutions to problems; e.g., *I consider multiple options before making a decision*; *I find it troublesome that there are so many different ways to deal with a difficult situation*) and 2) Control (the tendency to perceive difficult situations as controllable; e.g., *When I encounter difficult situations*, *I just don’t know what to do*; *I am capable of overcoming the difficulties in life that I face*). Responses to the items are measured on a seven-point Likert scale (*strongly disagree* to *strongly agree*). Items were reverse scored when necessary and summed. Higher scores indicated greater cognitive flexibility (Alternatives: α = 0.91; Control: α = 0.89).

### Data processing and analytic plan

**Fig 1:**
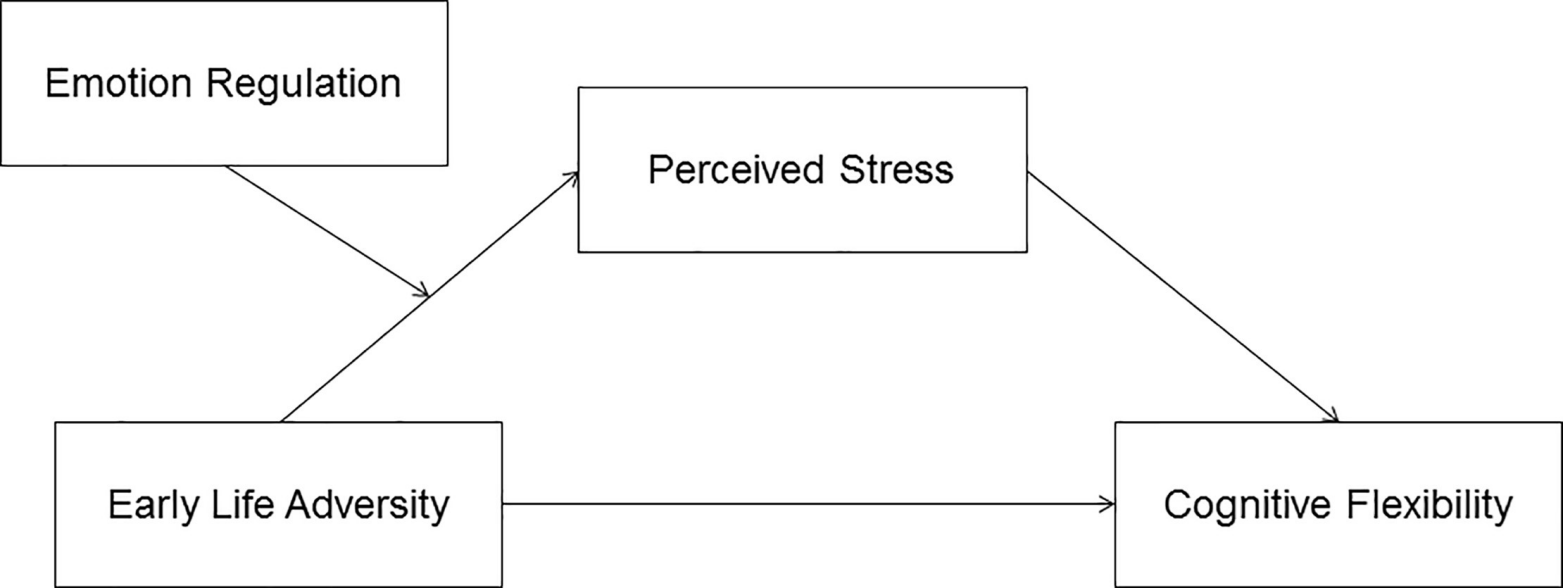
Moderated mediation model being tested using Hayes’ PROCESS model 7.

All variables were normally distributed (skew coefficient < |1|; kurtosis coefficient < |2|). In order to examine associations between ACEs, perceived stress, cognitive flexibility, and emotion regulation, we conducted bivariate correlations. Since our primary predictions were that ACEs would predict reduced cognitive flexibility and that perceived stress would mediate the relationship between ACEs and cognitive flexibility, we conducted a series of regressions with ACEs as the predictor variable, cognitive flexibility as the outcome variable and perceived stress as the mediator. Additionally, we hypothesized that emotion regulation strategies, either expressive suppression or cognitive reappraisal, would moderate the relationship between ACEs and perceived. See Fig 1. Therefore, we conducted two separate sets of regression analyses with each emotion regulation strategy, ACEs and their interaction as predictor variables and perceived stress as the dependent variable.

In order to conduct these analyses, we used Hayes’ PROCESS 3 macro model 7, which identifies these sets of regressions as moderated mediation analyses [55, 56], in SPSS version 25.0. The Hayes method uses boot-strapping and ordinary least-squares regression-based analyses to separately test the moderating and mediating paths of the hypothesized model. Then, parameter estimates from those tests are combined to test the moderated mediation, or the degree to which the mediation effect differs based on levels of the moderator [57]. This analytic strategy would ultimately help identify the degree to which the indirect effect of early adversity on cognitive flexibility through chronic stress is altered depending on emotion regulation strategy use.

## Results

Table 1 presents descriptive statistics and bivariate correlations of the variables. Our sample had a majority of participants (73.2%) who had experienced at least one ACE, while 42.1% of the sample reported four or more ACEs. Since CFI-Alternatives was not correlated with reported number of ACEs, we did not conduct a mediation analysis with this variable.

**Table 1:** Bivariate correlations and descriptive statistics.

N = 486	1.	2.	3.	4.	5.	6.	7.	8.
1. ACEs	-							
2. Cognitive Reappraisal	.03	-						
3. Expressive Suppression	.27***	.13**	-					
4. Perceived Stress	.37***	-.27***	.34***	-				
5. CFI Alternatives	-.04	.62***	.12**	-.20***	-			
6. CFI Control	-.43***	.12*	-.44***	-.74***	.15**	-		
7. Age	-.12*	.05	-.14**	-.19***	.09	.21***	-	
8. Education	.13*	.04	.18**	.12*	.04	-.20***	-.03	-
*Mean*	3.33	29.90	17.48	27.29	67.91	29.19	33.10	3.94
*SD*	3.23	6.34	5.22	7.19	11.79	9.38	9.66	1.06

* *p* < .05

** *p* < .01

*** *p* < .001; *Education levels*: *1 = some high school; 6 = graduate degree (Master’s*, *PhD*, *MD*, *etc*.*)*.

We sought to test two moderated mediation models in which reported number of ACEs both directly and indirectly, via perceived stress scores, predicted participants’ CFI-Control. In the first moderated mediation model, we tested whether the path from number of ACEs to perceived stress was moderated by individual differences in habitual use of cognitive reappraisal to regulate emotions. In the second moderated mediation model, we tested whether the path from ACEs to perceived stress was moderated by individual differences in habitual use of expressive suppression to regulate emotions.

Since prior research has shown that emotion regulation strategies vary with age [58, 59] and ACEs are associated with difficulties in school [60], we added participant age and education levels as covariates in both the models. All relevant variables were centered prior to analyses. Because a substantial number of participants (N = 77) chose not to report age, they were not included in the following analyses, leaving a final sample of 409. However, individuals who did and did not report their age did not differ significantly in their responses to any of the scales included in the models.

### Moderation

#### Cognitive reappraisal as a moderator

**Fig 2:**
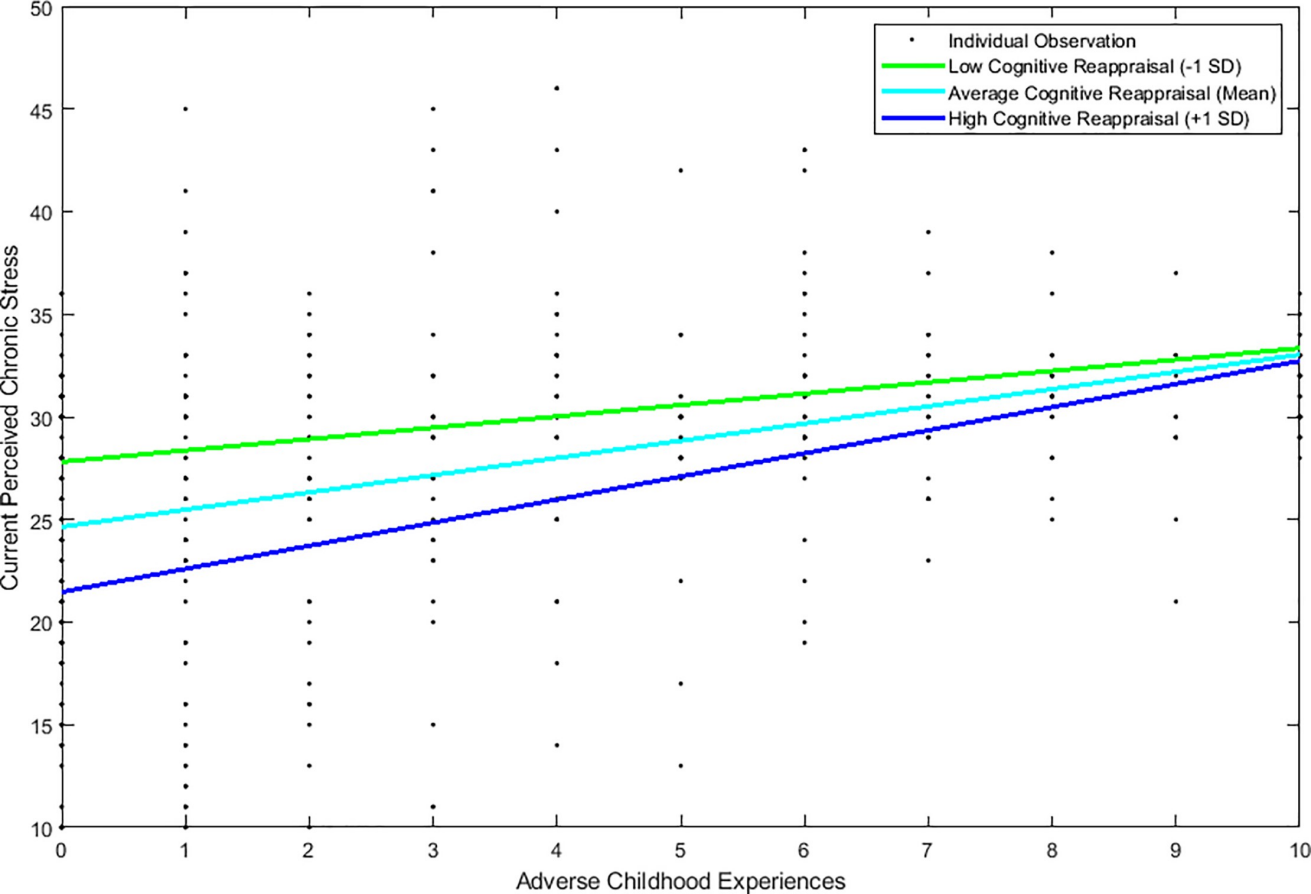
Relation between ACEs and PSS at high, average, and low levels of cognitive reappraisal.

Tests of moderation showed that the interaction between ACEs and cognitive reappraisal significantly predicted perceived stress when controlling for age and education, *R*^2^ = .27, *F*(5, 403) = 30.33, *p* < .001. Both ACEs, *B* = 0.79, *t*(403) = 8.18, *p* < .001, and reappraisal, *B* = -0.34, *t*(403) = -7.04, *p* < .001, were significantly associated with PSS. The interaction between ACEs and reappraisal was significantly associated with PSS, *B* = 0.04, *t*(403) = 2.36, *p* = .019. The relationship between ACEs and PSS scores was significant at one standard deviation above, *B* = 1.04, *t*(403) = 7.77, *p* < .001, and below the mean of cognitive reappraisal scores, *B* = 0.54, *t*(403) = 3.60, *p* < .001, as well as at the mean, *B* = 0.79, *t*(403) = 8.18, *p* < .001 (See Fig 2).

This suggests that, when reporting the same number of ACEs, individuals who used cognitive reappraisal more habitually reported less perceived chronic stress than those who used cognitive reappraisal less frequently. However, the buffering effect of cognitive reappraisal becomes less robust with the greater number of ACEs an individual reported. Thus, individuals with the lowest number of ACEs experienced more benefit from using cognitive reappraisal to regulate their emotions than those with the highest number of ACEs. Further, we used the Johnson-Neyman technique [61] with uncentered scores, which indicates that individuals who reported cognitive reappraisal scores below 19.7 (*M* = 29.90) showed no conditional effect of ACEs on perceived stress. Only 6% (~ 24 individuals) of the present sample falls below this threshold. These individuals showed consistently high perceived stress regardless of the number of ACEs.

#### Expressive suppression as a moderator

**Fig 3:**
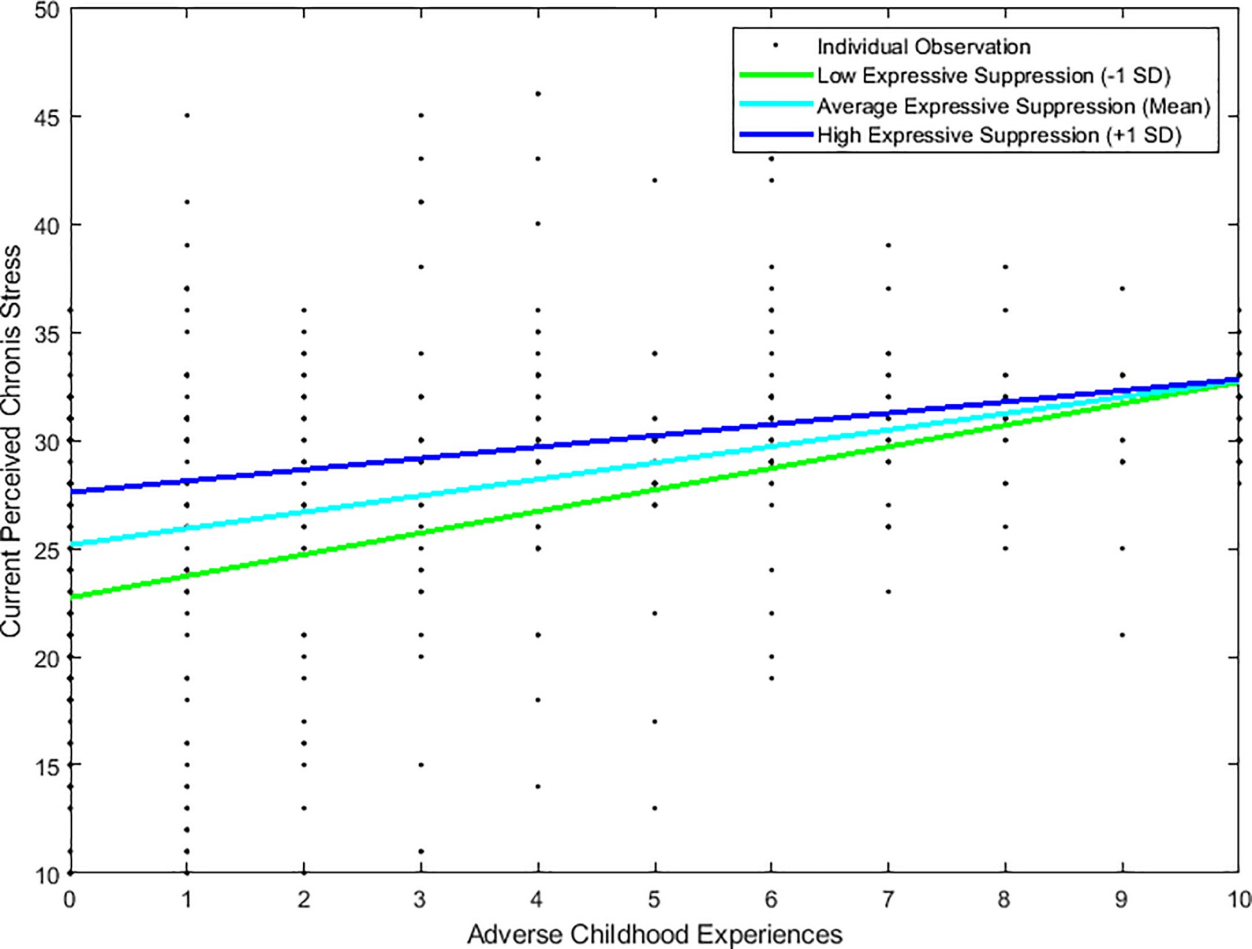
Relation between ACEs and PSS at high, average, and low levels of expressive suppression.

The test of the moderating effects of expressive suppression suggests that expressive suppression is also a significant moderator of the effect of ACEs on perceived stress, *R*^2^ = .23, *F*(5, 403) = 23.57, *p* < .001. Both the main effects of ACEs, *B* = 0.72, *t*(403) = 6.74, *p* < .001, and suppression, *B* = 0.29, *t*(403) = 4.38, *p* < .001, were significant and both coefficients were positive. Thus, our data suggest that both suppression and ACEs appear to be associated with greater perceived stress. Additionally, the interaction between ACEs and suppression was significantly associated with PSS, *B* = -0.04, *t*(403) = -2.07, *p* = .039. However, age was associated with reduced perceived stress, *B* = -0.09, *t*(403) = -2.61, *p* = .009. The relationship between ACEs and PSS scores was significant at one standard deviation above, *B* = 0.49, *t*(403) = 3.76, *p* < .001, and below the mean of expressive suppression scores, *B* = 0.96, *t*(403) = 5.46, *p* < .001, as well as at the mean, *B* = 0.72, *t*(403) = 6.73, *p* < .001 (See Fig 3).

This suggests that, in individuals reporting the same number of ACEs, those who used expressive suppression with greater frequency also reported greater perceived chronic stress than those who use expressive suppression less frequently. However, the effect of expressive suppression reduced as the number of ACEs an individual experienced increased. In other words, those with the fewest ACEs experienced the negative effects of expressive suppression more than those with the most ACEs. Further, we used the Johnson-Neyman technique [61] on uncentered scores, and discovered that individuals who reported levels of expressive suppression above 25.7 (*M* = 17.48) showed no conditional effect of ACEs on perceived stress. Approximately 4% (~16 individuals) of the sample fell above this threshold. These individuals reported high levels of perceived stress regardless of the ACEs score.

### Moderated mediation

When testing the mediating role of perceived stress in the relation between ACEs and CFI-Control, we found that ACEs, PSS, and the covariates accounted for 59% of the variance in CFI-Control scores, *R*^2^ = .59, *F*(4, 404) = 145.46, *p* < .001. PSS emerged as a significant predictor of CFI-Control, *B* = - 0.87, *t*(404) = -19.00, *p* < .001. However, the effect of ACEs remained significant when PSS was included in the model, *B* = -0.41, *t*(404) = -4.03, *p* < .001. However, because emotion regulation strategy use moderated the effect of ACEs on PSS, tests of the indirect effect will be conducted using moderated mediation analyses, as the indirect effect may be conditional upon values of the moderator.

#### CFI control model with cognitive reappraisal as moderator (See Fig 4)

This indirect effect of ACEs on CFI-Control through PSS scores was significant at all low, 95% CI: [-0.73, -0.21], average, 95% CI: [-0.85, -0.53] and high, 95% CI: [-1.13, -0.68] levels of reappraisal. Once again, these results indicate that a greater number of ACEs is associated with higher perceived chronic stress, which is in turn associated with a lessened sense of control in difficult life circumstances. Despite the significant indirect effect, it is noted that there remains a relationship between ACEs to CFI-Control, despite inclusion of the mediator. The index of moderated mediation is the formal test of moderated mediation in PROCESS which uses boot-strapping procedures to test the degree to which the indirect effects of the mediation model differ from each other based on different levels of the moderator [62]. The mediating effect of perceived stress in the relation between ACEs and CFI-Control is significantly moderated by cognitive reappraisal, as the confidence interval of the index of moderated mediation does not contain zero, *b* = -0.03, *SE* = .015, 95% CI: [-.064, -.004]. These results indicate that a greater number of ACEs is associated with higher perceived chronic stress, which is in turn associated with a lessened sense of control in difficult life circumstances. However, the relation between ACEs and perceived chronic stress is buffered by greater habitual use of cognitive reappraisal.

**Fig 4:**
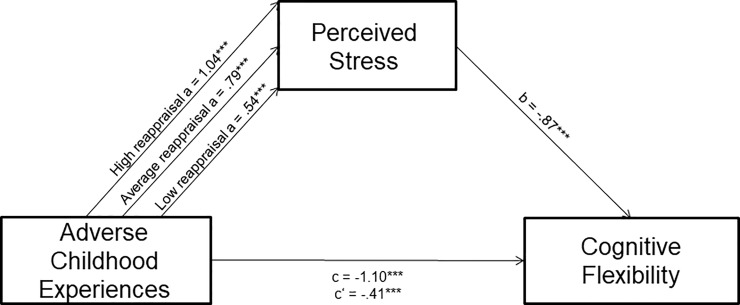
Cognitive reappraisal moderates the relationship between ACEs and perceived chronic stress, which is in associated with cognitive flexibility. Path *a* coefficients are displayed for high reappraisal (1 SD above the mean), average reappraisal (mean), and low reappraisal (1 SD below the mean).

#### CFI control model with expressive suppression as moderator (See Fig 5)

The indirect effect of ACEs Scores on CFI-Control through perceived stress scores was significant at low, 95% CI: [-1.13, -0.54], average, 95% CI: [-0.81, -0.46], and high, 95% CI: [-0.64, -0.22] levels of suppression. Additionally, the confidence interval of index of moderated mediation does not contain zero, suggesting the indirect effect of ACEs on CFI through PSS score is significantly moderated by suppression, *B* = 0.04, *SE* = 0.02, 95% CI: [.004, .075]. However, the relation between ACEs and perceived chronic stress is strengthened when greater habitual use of expressive suppression is used.

**Fig 5:**
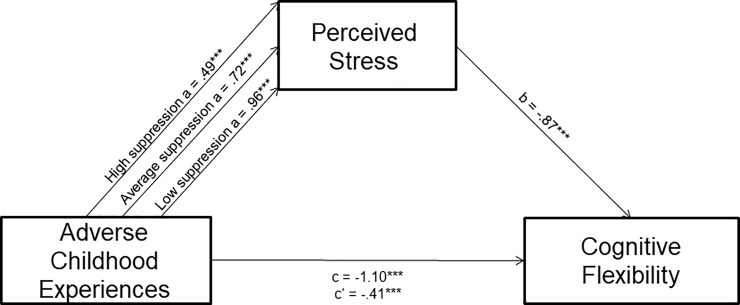
Expressive suppression moderates the relationship between ACEs and perceived chronic stress, which is in associated with cognitive flexibility. Path *a* coefficients are displayed for high suppression (1 SD above the mean), average suppression (mean), and low suppression (1 SD below the mean).

## Discussion

Approximately 70% of adults in North America report exposure to at least one adverse childhood experience (ACEs) [49]. Multiple studies have identified ACEs as a risk factor for mental health problems [63]. Yet, the impact of ACEs on psychological variables, such as emotion regulation and cognitive flexibility, are understudied. We conducted a study to examine the relationship between ACEs, perceived chronic stress, cognitive flexibility, and habitual use of emotion regulation strategies (reappraisal and suppression). To characterize the relationships between these variables, we conducted two moderated mediation models with CFI-Control as the key outcome variable, ACEs as the primary predictor variable, and perceived chronic stress serving as a mediator.

In the first model, we observed that number of ACEs predicted lower scores on CFI-Control. Specifically, exposure to ELA as measured by the ACEs had a significantly negative impact on participants’ CFI-Control. Since CFI-Control provides an indicator of the individual’s tendency to view difficult things as controllable, we believe this finding suggests that individuals exposed to ACEs are less likely to approach everyday difficulties in their life as controllable. We view this tendency to appraise all manner of difficulties as uncontrollable to be an indicator of inflexible appraisal of stressors in their environment as threatening, rather than challenging [64].

Additionally, we observed that the relationship between ACEs and CFI-Control was partially mediated by perceived chronic stress. In essence, the negative impact of ACEs on cognitive flexibility was both direct and through perceived stress levels. These analyses indicate that individuals with higher number ACEs are more likely to report experiencing stress, which in turn, also reduces their ability to flexibly appraise environmental demands. Thus, our data suggest that this inflexible appraisal of environmental challenges is partially driven by individuals feeling taxed and overwhelmed by circumstances [64]. Concordant with previous reports, our data demonstrate a dosage effect [3], such that the higher the number of ACEs a person reported the more likely they were to report feeling stressed and that they could not cope with challenges.

Finally, we found that individual differences in the habitual use of reappraisal to regulate emotions moderates the relationship between ACEs and perceived chronic stress. Specifically, our data indicated that habitual use of reappraisal attenuated the effect of ACEs on perceived chronic stress. Individuals who habitually regulated their emotions by reappraising were also less chronically stressed. Consequently, the interactive effect of reappraisal also influenced an individual’s inflexible appraisal of environmental demands. Our data add support to other research showing that adaptive emotion regulation strategies can buffer against the effects of ELA [14].

In the second model, we observed the same the relationships between ACEs, perceived chronic stress, and CFI-Control as the first model. That is, higher number of ACEs predicted lower scores on CFI-Control. This relationship was partially mediated by perceived stress levels. Additionally, habitual use of expressive suppression in conjunction with number of ACEs influenced individual’s perceived stress. The data indicated that, in individuals with the same number of ACEs, habitual use of expressive suppression was more likely to result in increased chronic stress. This finding provides support for the notion that that use of expressive suppression as an emotion regulation strategy intensifies the effect of ELA on psychological outcomes [65]. Since chronic stress is associated with negative health outcomes, our data suggest that habitually using expressive suppression may not be a beneficial emotion regulation strategy for individuals with ELA. Our work adds to support to the notion that habitual use of expressive suppression is associated with negative psychological outcomes [20].

It is important to note that for those individuals who had very high number of ACEs the interactive effect of cognitive reappraisal or expressive suppression on perceived chronic stress was muted (see Figs 2 and 3). Our data are consistent with prior work showing that, in individuals who have experienced very high levels of early adversity, psychological functioning may be determined by factors other than emotion regulation [66]. As indicated in Fig 2, individuals with 10 ACEs who also report high habitual use of cognitive reappraisal exhibited chronic stress levels that were higher than individuals with 0 ACEs and low frequency of reappraisal to regulate emotions. In contrast, as shown in Fig 3, individuals with 10 ACEs reported similar levels of chronic stress regardless of high or low frequency of expressive suppression to regulate emotions. This finding further highlights that the impact of ACEs accumulates with increasing exposure to different types of adversities [3, 63]. Since ACEs are a factor that cannot be modified, future research should consider that individuals with very high number of ACEs might be potential targets for treatments aimed at improving emotion regulation skills. In view of the fact that cumulative trauma in early development is associated with higher levels of psychopathology in adulthood (e.g., substance abuse [3]), prevention programs that focus on the improving emotion regulation skills in children and young adults may be able to counter some of the negative effects of early adversity [66].

The difference in the moderating effect of expressive suppression and reappraisal could be the result of two related reasons. First, expressive suppression is a response-focused strategy that emerges once the emotional event is underway and requires constant monitoring resources to maintain. This can make it cognitively costly [67, 68], thereby leaving fewer cognitive resources for dealing with environmental demands. Thus, the observed reduction in cognitive flexibility could be a by-product of reduced cognitive resources overall. Second, expressive suppression can have a degrading effect on the individual’s relationships and social functioning by negatively impacting contingent responsiveness in social interactions [69]. Given that ACEs are associated with greater interpersonal difficulty [63], it is possible that use of expressive suppression as an emotion regulation strategy makes individuals feel unsupported and alone when confronting difficulties. As a result, expressive suppression serves to enhance inflexible appraisal of stressors as threats. Regardless, our data add further credence to the notion that habitual use of expressive suppression may be maladaptive [20].

One possible explanation for perceived chronic stress emerging as a mediator between ACEs and CFI-Control is that individuals with high number of ACEs do have more stressors in their life. For instance, past research has shown that ACEs are linked with poor economic outcomes and food insecurity [70]. It is relevant to note that we controlled for education levels in our analyses. However, it is also possible that the inflexible appraisal of stressors we observe in participants with high ACEs scores is due to enhanced development of vigilance to threats in the environment [7]. This might foster attention to or monitoring of stressors [71], but appears to come at the cost of flexibility. Overall, our data add some support to the notion that exposure to early abuse alters threat appraisal in adulthood [7].

Interestingly, we did not observe an association between ACEs and CFI-Alternatives. This provides further evidence that the two subscales of the CFI assess different aspects of cognitive flexibility [34]. In the original report on the development of the instrument, it was CFI-Control that had exhibited larger correlations with depression scores across two studies (*r* = -.50; *p* = .001 and *r* = -.44; *p* = .001) compared to CFI-Alternatives (*r* = -.19; *p* = .01 and *r* = -.20; *p* = .01). Thus, it is possible that CFI-Control is more likely to be associated with outcomes than CFI-Alternatives. Considering that CFI-Alternatives is an indicator of an individual’s ability to view a problem from multiple perspectives, our data suggest that ACEs do not influence this aspect of cognitive flexibility. This is a hopeful and encouraging finding, particularly for individuals with exposure to ELA. Although empirical evidence suggests that early life stress is associated with reduced cognitive flexibility in children and adolescents [41, 44], our data indicate that ELA might not influence all aspects of cognitive flexibility in adults.

The fact that our sample size had a wide age range (19–86 years) may have influenced our results. For instance, participant age was positively associated with CFI-Control and negatively associated with perceived stress and habitual use of suppression as a strategy to regulate emotions. Although we controlled for age effects in our analyses, our data suggest that studies that systematically examine the effect of ACEs on different aspects of cognitive flexibility across the life span are warranted. Considering that this relationship remains significant after accounting for variance explained by perceived stress levels, participant age, and education levels, our study highlights the significance of ACEs in predicting inflexibility.

Several limitations must be noted when interpreting our findings. First, our work is based on self-report measures. There is some evidence to suggest that behavioral and self-report measures provide insight about different aspects of cognitive flexibility [36], so it is possible that the observed associations would differ if a behavioral measure of cognitive flexibility (e.g., WCST) were used. Second, we did not collect data on participants’ current or past economic status. This is a major confound that precludes us from differentiating between the effects of economic hardship and physical and psychological trauma in development. Childhood poverty is accompanied by psychological and material hardships that profoundly impact the trajectory of development [12]. We made this decision prior to data collection based on previous studies with ACEs showing that education, not income, was associated with outcomes [3]. However, we also acknowledge that the sample used in those studies consisted of majority white, middle class, and educated individuals [72]. Therefore, it is entirely possible that individuals who are living in economic deprivation are also experiencing increased feelings of helplessness which can be exhibited as reduced control over challenging circumstances. Since empirical evidence has suggested that increased levels of adversity exist in lower income families [72], future research should include socioeconomic status in their study design. Third, the proposed mediation model may not extend to populations with mental health problems [73]. Despite empirical evidence from rodent models [74], the observed direction of the relation between perceived stress and cognitive flexibility should ideally be resolved through longitudinal studies. Our study design was correlational and needs to be replicated before any firm conclusions can be drawn. Finally, we did not randomize the order in which the questionnaires were presented. Nevertheless, by studying cognitive flexibility as an outcome variable in adults, we were able to extend the existing literature on ACEs. Additionally, by investigating the moderating effect of emotion regulation strategies, our study provides further evidence that effective emotion regulation can be a key resilience factor in individuals who have experienced early adversity.
